# A Functional Yeast-Based Screen Identifies the Host Microtubule Cytoskeleton as a Target of Numerous *Chlamydia pneumoniae* Proteins

**DOI:** 10.3390/ijms24087618

**Published:** 2023-04-20

**Authors:** Carolin Wevers, Mona Höhler, Abel R. Alcázar-Román, Johannes H. Hegemann, Ursula Fleig

**Affiliations:** 1 Eukaryotic Microbiology, Institute of Functional Microbial Genomics, Heinrich-Heine-University, 40225 Düsseldorf, Germany; 2 Institute of Functional Microbial Genomics, Heinrich-Heine-University, 40225 Düsseldorf, Germany

**Keywords:** microtubule cytoskeleton, *Schizosaccharomyces pombe*, yeast, effector proteins, screen, *Chlamydia pneumoniae*, MAPs, pathogen, bacteria

## Abstract

Bacterial pathogens have evolved intricate ways to manipulate the host to support infection. Here, we systematically assessed the importance of the microtubule cytoskeleton for infection by *Chlamydiae*, which are obligate intracellular bacteria that are of great importance for human health. The elimination of microtubules in human HEp-2 cells prior to *C. pneumoniae* infection profoundly attenuated the infection efficiency, demonstrating the need for microtubules for the early infection processes. To identify microtubule-modulating *C. pneumoniae* proteins, a screen in the model yeast *Schizosaccharomyces pombe* was performed. Unexpectedly, among 116 selected chlamydial proteins, more than 10%, namely, 13 proteins, massively altered the yeast interphase microtubule cytoskeleton. With two exceptions, these proteins were predicted to be inclusion membrane proteins. As proof of principle, we selected the conserved CPn0443 protein, which caused massive microtubule instability in yeast, for further analysis. CPn0443 bound and bundled microtubules in vitro and co-localized partially with microtubules in vivo in yeast and human cells. Furthermore, CPn0443-transfected U2OS cells had a significantly reduced infection rate by *C. pneumoniae* EBs. Thus, our yeast screen identified numerous proteins encoded using the highly reduced *C. pneumoniae* genome that modulated microtubule dynamics. Hijacking of the host microtubule cytoskeleton must be a vital part of chlamydial infection.

## 1. Introduction

Obligate intracellular pathogens rely on intensive molecular crosstalk with their eukaryotic host cells. The compartmentalization of the host cell requires an elaborate cytoskeletal network that consists of the F-actin and microtubule (MT) cytoskeleton, intermediate filaments and septins. These structures feed into numerous cellular processes, including the maintenance of the cell shape and mobility, vesicle transport via motor proteins, endo- and exocytosis, organization, subcellular positioning and interconnection of organelles [1,2,3,4,5]. Thus, for obligate intracellular pathogens with an intracellular developmental cycle within a membrane-bound compartment, the rearrangement and repurposing of host cellular structures, such as the dynamic host cytoskeleton is required. While the modulation of the host actin cytoskeleton by bacterial pathogens is well studied (for reviews, see [6,7,8]), our knowledge of how the MT cytoskeleton is used and altered by pathogenic bacteria is scarce (for a review, see [9]). A few bacterial effector proteins were identified that alter MT dynamics, such as VirA from *Shigella* and EspG from *E. coli* (for a review, see [9]). However, their MT-modulating function during the infection process is unclear.

The obligate intracellular Gram-negative *Chlamydiae* are responsible for several serious diseases in humans worldwide. *Chlamydia pneumoniae* (*Cpn*) infects the respiratory tract and its infection is associated with chronic diseases, including Alzheimer’s, atherosclerosis, asthma and lung cancer (for reviews, see [10,11,12]). In contrast, *Chlamydia trachomatis* (*Ctr*) causes serovar-dependent sexually transmitted infections of the urogenital tract and severe eye infections (for reviews, see [13,14]). All *Chlamydiae* have a unique biphasic developmental cycle in a parasitophorous membrane-bound vesicle called inclusion. An infection starts with the extracellular infectious elementary body (EB) attaching to and activating receptors on target cells. In parallel, effector proteins are secreted via the type III secretion system (T3SS) targeting host cell processes and structures for EB uptake [15]. After internalization, EBs differentiate into non-infectious, metabolically active reticulate bodies (RB), followed by rounds of replication via binary fission, before RBs re-differentiate back to EBs and are released by host cell lysis or extrusion [15,16]. For a successful chlamydial infection, the manipulation of and interaction with host cytoskeletal elements is essential. The analysis to date has almost exclusively focused on the *Ctr* infection process, where global disruption of F-actin structures prior to the infection strongly reduced the entry of *Ctr* serovar L2 EBs into mammalian cells [17,18,19]. In contrast, the role of the MT cytoskeleton for *Ctr* host cell entry is less clear. Disruption of MT structures via vinca alkaloids prior to and during incubation with *Ctr* L2 EBs attenuated the subsequent infection [20], while MT depolymerization via nocodazole did not reduce *Ctr* serovars L2 and E host cell entry [18]. Later during infection, MTs are used as tracks for *Ctr* inclusions to move to the MT-organizing center (MTOC), where the inclusion resides and inclusion fusion occurs [18,21,22]. During the replicative phase, the growing *Ctr* inclusion is encaged by F-actin, intermediate filaments and septins [23,24]. At the mid and late stages of development, stabilized MTs are present at the periphery of the inclusion and their disruption reduces the formation of infectious progeny [25].

To date, many of the chlamydial effector proteins interacting with and modulating cytoskeletal structures during the infection cycle remain elusive. The actin cytoskeleton is targeted via the secreted *Ctr* effectors TarP (and its *Cpn* homolog CPn0572) and TmeA during EB entry [26,27,28,29]. Transport of the early *Ctr* inclusion to the MTOC is mediated by binding of the inclusion membrane protein (Inc protein) Ct850 to the dynein light chain DYNLT1 [30]. Ct850 also interacts with the inclusion membrane protein Ct222, but its role in transport to the MTOC is not known [31]. Interestingly, during a *C. psittaci* infection, the inclusion membrane protein IncB binds to the dynein-interacting human protein Snapin, thus establishing a second connection between MTs and a chlamydial inclusion [32]. MT assembly around the *Ctr* inclusion requires the inclusion membrane protein Ct223/IPAM and the host centrosomal protein CEP170 [33]. Moreover, inclusion integrity involves the inclusion membrane protein InaC, which controls infection via activation of the host cell GTPases ARF1 and RhoA formation and dynamics of both the actin and MT scaffolds surrounding the *Ctr* inclusion [34,35]. Finally, the Inc protein CT192 is necessary for dynactin recruitment to the inclusion membrane [36]. In general, chlamydial effector proteins targeting the cytoskeleton require secretion into the host cytosol, where they target host cell proteins of interest, or localization at the chlamydia–host cell interface, in the inclusion membrane. Translocation of effector proteins into the host cytosol and the inclusion membrane is probably mediated by the type 3 secretion system (T3SS), which is a form of macromolecular machinery that transports proteins from the bacterial cytosol directly through the inner and outer bacterial membranes and through the inclusion membrane. Inc proteins are embedded in the inclusion membrane and are unique to *Chlamydiaceae*. The incorporation of Inc proteins into the inclusion membrane, which was derived from the plasma membrane due to the EB internalization process, leads to an extensive modification of the inclusion membrane. Inc proteins are characterized by one or more hydrophobic regions and possess a uni-directional hairpin-like topology in the inclusion membrane, thus decorating the cytosolic face of the inclusion membrane with their N- and C-termini and, in this way, drive the interaction of the chlamydial organelle with the host cell [37]. Several Incs show some moderate conservation between different *Chlamydiaceae* species, but each chlamydial species also has several unique Incs [38]. Using Inc-specific criteria, for *Cpn*, a total of 107 Inc proteins were predicted, almost all of which are functionally unexplored [39].

In *Cpn*, only a single protein was identified that modulates MTs. The evolutionarily conserved T33S effector protein CopN is an MT destabilizer in vitro but its role during infection is unknown [40,41]. Thus, we employed *Cpn* to assess (i) the impact of the host MT cytoskeleton on the early processes of chlamydial infection and (ii) the importance of the MT cytoskeleton during *Cpn* infection. As the obligate intracellular bacteria are very difficult to manipulate genetically, we used the fission yeast *Schizosaccharomyces pombe* as a screening tool. Determination of alterations of yeast interphase MT upon the expression of chlamydial genes uncovered the existence of numerous chlamydial proteins involved in host MT manipulation. Thus, the importance of the host MT cytoskeleton in chlamydial infection was found to be vastly underestimated.

## 2. Results

### 2.1. Early Steps in Cpn Infection Required an Intact MT Cytoskeleton

**Figure 1:**
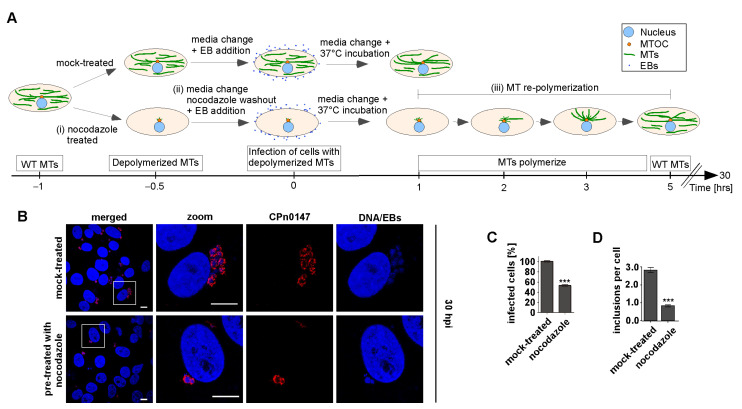
The host MT cytoskeleton was required for chlamydial infection. (**A**) Schematic representation of the experimental setup used: mammalian HEp-2 cells were mock-treated (DMSO-treated, top panels) or treated with the MT-destabilizing drug nocodazole (bottom panels) prior to infection with purified *Cpn* EBs (MOI = 5) for 30 h. Nocodazole treatment (bottom panels) was as follows: (i) cells were incubated with nocodazole for 30 min at 37 °C and MT depolymerization was monitored microscopically. (ii) Nocodazole washout via a media change was followed by infection with *Cpn* EBs. (iii) Repolymerization of MTs was monitored (Figure S1) and was complete after 5 h. (**B**) Representative confocal images of mock-treated or nocodazole-treated HEp-2 cells infected with purified EBs. Chlamydial inclusions (red) were visualized with an anti-CPn0147 antibody (chlamydial inclusion membrane protein) and DNA (blue) with DAPI. White boxes show enlargements (zoom). Scale bars: 10 µm. (**C**) Quantification of the number of infected HEp-2 cells. Treatment of cells with/without nocodazole prior to the infection depicted in (**A**). (**D**) Quantification of the number of chlamydial inclusions per infected HEp-2 cell that were treated with/without nocodazole prior to infection. (**C**,**D**) three independent experiments/per condition as described in (**A**), each with 100 cells; error bars represent ± SEM; *t*-test: *p* < 0.001 (***).

The role of the host cell MT cytoskeleton in the *Cpn* infection cycle had not yet been studied. Therefore, we first used an assay to determine whether an intact MT cytoskeleton was required in the early steps of the infection. We reasoned that destabilizing MTs at a later time point in the infection process would have severe consequences for host cell functions, and thus, we only assayed infection in cells with no MTs prior to the infection. Human epithelial HEp-2 cells were treated with the MT-destabilizing drug nocodazole prior to a *Cpn* infection. As determined using microscopy, MTs were fully depolymerized before infection (Figure 1A and Figure S1). The washout of nocodazole with the simultaneous addition of *Cpn* EBs resulted in a scenario where EBs infected the human cells in the absence of the MT network. The MT network repolymerized within the next 5 h (Figure S1). Epithelial cells treated in this way showed a 46% reduction in *Cpn*-infected cells in comparison to mock-treated control cells (Figure 1B,C). Furthermore, the number of inclusions per cell decreased in the nocodazole-treated cells in comparison to the mock-treated cells (Figure 1D). Thus, an intact interphase MT cytoskeleton was required for the early processes of *Cpn* infection.

### 2.2. Identification of Cpn MT-Modulating Proteins via the S. pombe Test System

To identify chlamydial proteins that modulate the MT cytoskeleton, we decided to use the fission yeast *Schizosaccharomyces pombe*, as fundamental features of the MT cytoskeleton are conserved and this yeast is an excellent model organism for the identification of MT-modulating proteins: it features an extensive interphase MT cytoskeleton organized in MT bundles whose dynamic behavior can be imaged and measured easily via life-cell imaging of α-tubulin-GFP [42,43,44].

**Figure 2:**
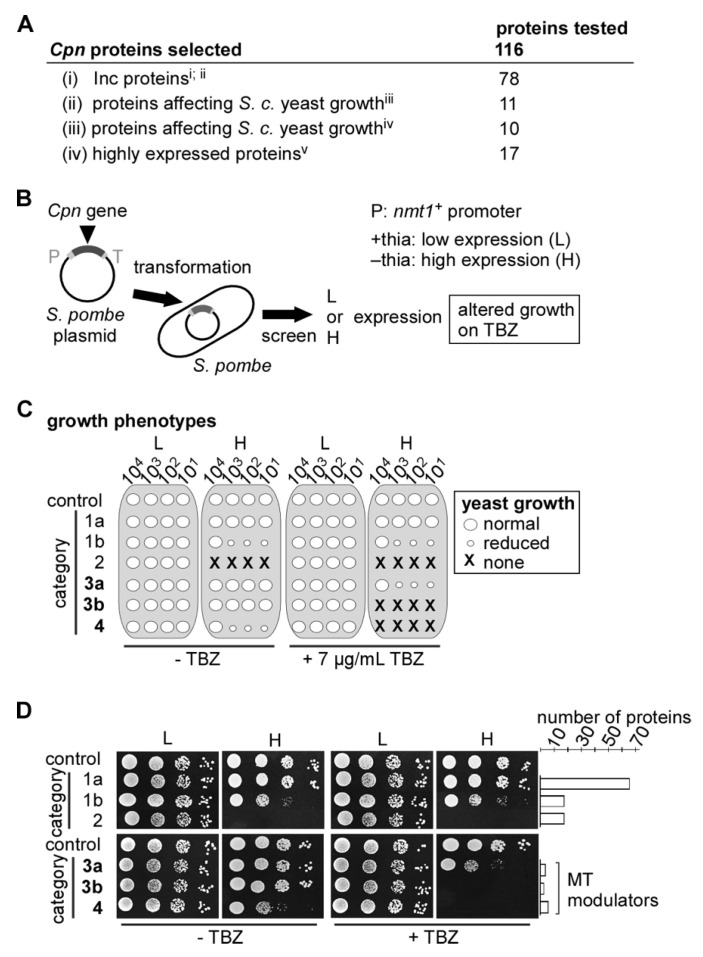
Identification of *Cpn* MT-modulating proteins via fission yeast. (**A**) The 116 *Cpn* proteins chosen for the analysis were selected from Inc proteins (i, [39]; ii, [45]), proteins that affect *S. cerevisiae* yeast growth (iii, [46]; iv, [47]) or those that are highly expressed in a transcriptome analysis (v, [48]). (**B**) Schematic representation of the screen setup. Each *Cpn* gene (dark grey) was cloned into an *S. pombe* plasmid flanked by the thiamine-repressible *S. pombe nmt1^+^* promoter (P) and *nmt1^+^* terminator (T). *S. pombe* wild-type cells transformed with such a plasmid were tested for growth defects in the presence of 7 μg/mL of the MT-destabilizing drug thiabendazole (TBZ). The presence of thiamine (+thia) resulted in low expression (L) from the *nmt1^+^* promotor, while no thiamine (-thia) resulted in high expression (H) from *nmt1^+^.* (**C**) Schematic representation of a serial dilution patch assay (10^4^–10^1^ cells) that showed all possible phenotypes scored by the expression of a chlamydial gene in *S. pombe*. Growth of transformants was compared with a transformant expressing a control plasmid (no chlamydial gene). Normal growth (similar to control transformant growth) (big circle), reduced growth (small circle) and no growth (X). (**D**) Examples of serial dilution patch tests of a control transformant and categories 1 to 4 shown in (**C**). Overall, 67/116 *Cpn* genes tested gave rise to yeast growth phenotype 1a, 18/116 to 1b, 18/116 to 2, 4/116 to 3a, 3/116 to 3b and 6/116 to category 4 phenotype. Sensitivity to TBZ was scored after 6 days at 25 °C. Chlamydial proteins that when expressed in yeast gave rise to category 3a, 3b or 4 phenotypes were chosen for further analysis.

Our screen to identify *Cpn* MT-modulating proteins encompassed the (i) selection of chlamydial genes to be assessed; (ii) cloning of these genes into an *S. pombe* expression vector and (iii) transformation of yeast with these plasmids and identification of transformants sensitive to MT poisons. In total, 116 genes from the evolutionarily streamlined *Cpn* (GiD) proteome of 1074 protein-coding ORFs were selected for the analysis (Figure 2A). These include 78 genes encoding putative inclusion membrane (Inc) proteins [39,45]. A total of 21 genes encoded proteins that led to a growth defect when expressed in the budding yeast *Saccharomyces cerevisiae* [46,47] and 17 genes coded for highly expressed proteins [48]. A list of all proteins tested is found in Table S1B. Chlamydial genes encoding these proteins were cloned into an *S. pombe* expression vector behind the repressible *S. pombe nmt1^+^* promoter. Low-level expression from this promoter is in the presence of thiamine, while high-level expression is in the absence of thiamine [49]. Transformants of a wild-type *S. pombe* strain expressing a chlamydial gene were then tested for reduced growth and increased sensitivity to the MT-destabilizing drug thiabendazole (TBZ) (Figure 2B).

A total of 67 chlamydial genes that were expressed either under low- or high-level conditions had no discernible effect on yeast growth (Figure 2C,D, category 1a), while the expression of a further 18 chlamydial genes resulted in a moderate growth defect but no increased TBZ sensitivity (Figure 2C,D, category 1b). The 18 chlamydial genes in category 2 were lethal to yeast cells under high gene expression and showed no increased sensitivity to TBZ under low expression (Figure 2C,D). Thus, genes in categories 1a, 1b and 2 had no measurable effect on the yeast MT cytoskeleton and were not analyzed further. A list of which protein was put into which category is shown in Table S1B.

The remaining 13 chlamydial genes all caused an increased sensitivity to TBZ when expressed in yeast. Depending on the severity of the phenotypes of the transformants, chlamydial genes were grouped into categories 3a, 3b and 4. Four genes caused reduced growth of the transformants on TBZ under high expression conditions (category 3a), while the expression of a further three resulted in lethality on TBZ-containing media (category 3b). Lastly, the expression of six chlamydial genes resulted in reduced growth on media without TBZ and lethality on media with TBZ (category 4) (Figure 2C,D). Thus, 13/116 chlamydial genes tested in our *S. pombe* assay gave rise to increased sensitivity to an MT poison, suggesting that they encoded chlamydial proteins that modulated the MT cytoskeleton of the host cell.

Of the 13 chlamydial proteins identified in our yeast screen, 12 had initially been predicted to be Inc proteins [39]. However, the Phobius prediction program showed that CPn0045 did not fit the criteria for Inc proteins (Figure S2), thus 11/13 proteins were likely to be Inc proteins. As shown in Figure S2, the 11 candidates shared the typical Inc structure with the N- and C-termini of each protein facing the host cytosol. CPn0821 had no transmembrane (TM) domain and was likely to be a soluble protein (Table S2). None of the 13 identified *Cpn* proteins has been characterized to date in relation to chlamydial infection. As shown in Table S2, 5/13 proteins, namely, CPn0065, CPn0312, CPn0443, CPn0565 and CPn0821, had a putative homolog in *Ctr* and other chlamydia, while 1/13, namely, CPn0186, had a homolog in *Ctr* only. The identity between homologs is limited (24% to 35% for full-length proteins). A total of 2/13 proteins, namely, CPn0357 and CPn0365, had homologs in other chlamydia but not in *Ctr*. The remaining five proteins, namely, CPn0045, CPn0216, CPn0284, CPn0372 and CPn1027, were unique to *Cpn* (Table S2).

### 2.3. S. pombe Tubulin Mutants Became Synthetically Lethal upon the Expression of Chlamydial Genes

**Figure 3:**
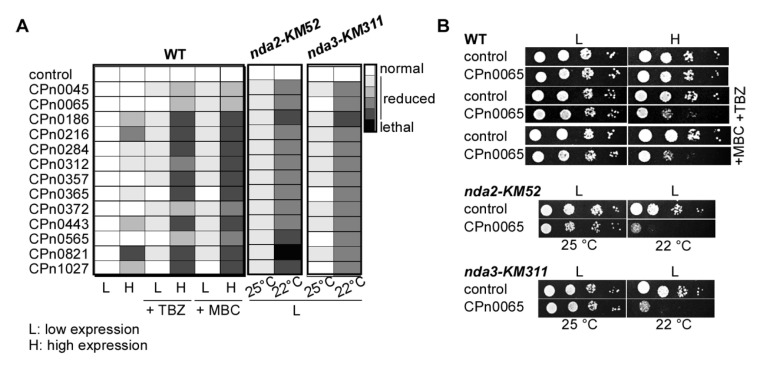
Expression of all 13 *Cpn* proteins in yeast resulted in MT hypersensitivity and synthetic lethality with tubulin mutants. (**A**) Growth phenotypes of *S. pombe* transformants that expressed one of the indicated 13 *Cpn* genes (systematic names on the left). The severity of the growth defect is indicated by the shades of the box (the darker, the more severe). The strains analyzed were the wild-type (WT) and the cold-sensitive *nda2-KM52* and *nda3-KM311* mutant strains. Media was with TBZ or MBC if indicated. Growth of WT was undertaken for 5 days at 25 °C. The tubulin mutant strains grew for six days at the indicated temperatures. Top panel in (**B**): *S. pombe* serial dilution patch tests (10^4^–10^1^ cells) of *cpn0065*-expressing WT cells grown under plasmid-selective conditions at 25 °C for 6 days without or with either 7 μg/mL TBZ or 2.5 µg/mL MBC; L: low expression, H: high expression. Middle and bottom panels: serial dilution patch tests of cold-sensitive *nda2-KM52* and *nda3-KM311* transformants that expressed *cpn0065* incubated at 25 °C or 22 °C for 6 days; L: low expression.

To further characterize the modulation of MTs by the 13 chlamydial proteins, we tested (i) whether yeast transformants expressing one of these genes were also sensitive to the MT inhibitor methyl benzimidazol-2-yl-carbamate (MBC) and (ii) whether the expression of these genes in conditional-lethal tubulin mutant strains led to synthetic lethality. In all cases, growth on the MBC-containing medium was similar to that observed for the TBZ-containing medium (summarized in Figure 3A and Figure S3A). Furthermore, and in contrast to the expression in wild-type cells, low-level expression of these chlamydial genes in the cold-sensitive α-tubulin *nda2-KM52* or β-tubulin *nda3-KM311* mutant strains [50] led to massive growth reduction at the semi-permissive temperature of 22 °C (Figure 3A,B and Figure S3B,C). High-level expression of all 13 chlamydial genes in these tubulin mutant strains was lethal under all conditions.

### 2.4. The S. pombe Interphase MT Cytoskeleton Became Highly Aberrant upon the Expression of Chlamydial Genes

To determine whether and how the 13 identified chlamydial proteins altered the *S. pombe* MT cytoskeleton, we used a GFP-α-tubulin strain to visualize MTs via life-cell imaging. This strain endogenously expresses the minor α-tubulin (*S. pombe* has two α-tubulin genes) fused to GFP via the weak *nmt81* promoter, which leads to slightly altered MT properties compared with a wild-type strain [51,52].

**Figure 4:**
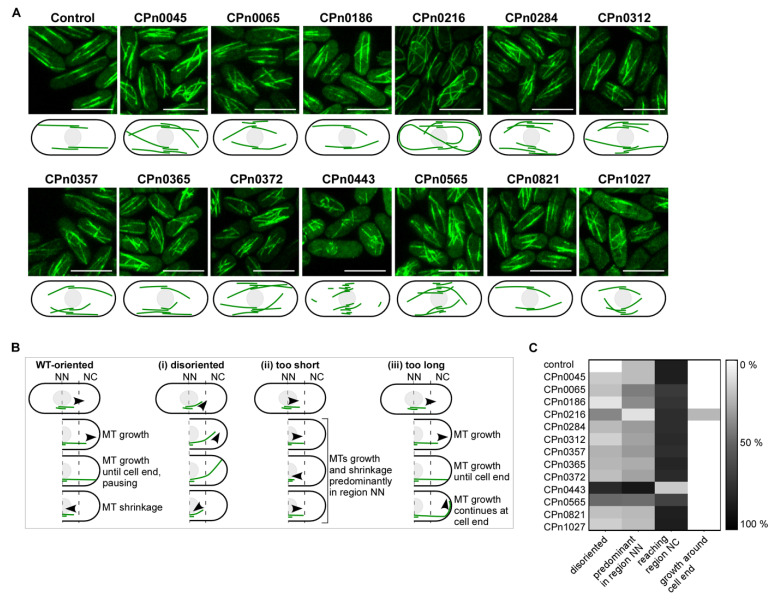
*Cpn* proteins cause massive alterations in the *S. pombe* interphase MT cytoskeleton. *S. pombe* cells that expressed *gfp-α-tubulin* (*nmt81::GFP-atb2^+^*) were transformed with a control plasmid or plasmids carrying one of the 13 *Cpn* genes highly expressed from the *nmt1^+^* promoter. (**A**) Photomicrographs (MTs in green) and the corresponding schematic representations. Scale bars: 10 µm. (**B**) Schematic illustration of WT interphase MT dynamics and the alterations observed for *Cpn* gene expressing cells. (i) Disoriented MTs, (ii) aberrantly short MTs or (iii) aberrantly long MTs. Black arrowheads show the directions moved for growing/depolymerizing MT. Cells were divided into 3 parts: the middle region was defined as being close to the nucleus (NN) and the other two regions as NC (regions close to either cell end). (**C**) Heatmap of the aberrant interphase MT phenotypes caused by *Cpn* proteins as described in (**B**). A total of 100 MTs/transformant strains were counted for disoriented MTs, MTs predominant in region NN only, MTs reaching the cell end (region NC) and MTs that continued to grow when the cell end had been reached (growth around cell end).

Expression of all 13 *Cpn* genes in this strain led to TBZ sensitivity (Figure S3D) and altered interphase MT organization (Figure 4A). In wild-type cells, the three to four polarized MT bundles present in the cytoplasm orientated along the long axis of the cell, extending with their MT-plus ends toward the cell end and their MT-minus ends overlapping at the cell center (control in Figure 4A, shown diagrammatically in Figure 4B). MT-plus ends reaching a cell tip will pause before depolymerizing back to the cell center [53,54].

All yeast transformants expressing one of the 13 chlamydial genes showed alterations of the MT cytoskeleton; some of them were severe (Figure 4A–C). The most prominent were MT bundles that were not aligned with the long axis of the cell, such as those seen for CPn0565 transformants, which were much shorter than wild-type MTs, as seen in transformants expressing CPn0443, or MT bundles that were much longer than those of the wild-type and curled around the cell tip, as observed for CPn0216 transformants (Figure 4A–C). To better define the MT phenotypes observed, we divided a cell into three parts and quantitatively determined via live-cell imaging the number of MTs in the middle part close to the nucleus, MT bundles that were not oriented along the long-axis of the cell and MT bundles that curled around the cell tip (Figure 4B). All transformants expressing a chlamydial gene featured misorientated MT bundles, but with the exception of CPn0443 transformants, such MT bundles were still able to reach the cell tip (Figure 4C). Transformants that expressed CPn0065, CPn0443 or CPn0565 all had abnormally short MTs. The CPn0443 phenotype was the most severe, with the vast majority of MTs never reaching the cell end (Figure 4A,C). Such short MTs are reminiscent of strains with a deletion of the *mal3^+^* gene [55]. Mal3 is a member of the plus-end-binding protein 1 (EB1) family that tracks MT-plus ends and is central to the regulation of MT dynamics [56]. In contrast, the expression of CPn0216 resulted in MTs that were aberrantly long, as MT plus-ends continued to grow upon reaching the cell tip (Figure 4C). Such a phenotype was observed in various mutants of MT-modulating proteins, such as components of the yeast MTOC or kinesin-8 motor proteins [57,58].

We concluded that all 13 chlamydial proteins altered the yeast MT interphase cytoskeleton, albeit in different ways, demonstrating that modulation of the host MT during a chlamydial infection must be manifold and diverse.

### 2.5. CPn0216 and CPn0443 Altered the MT Dynamics

**Figure 5:**
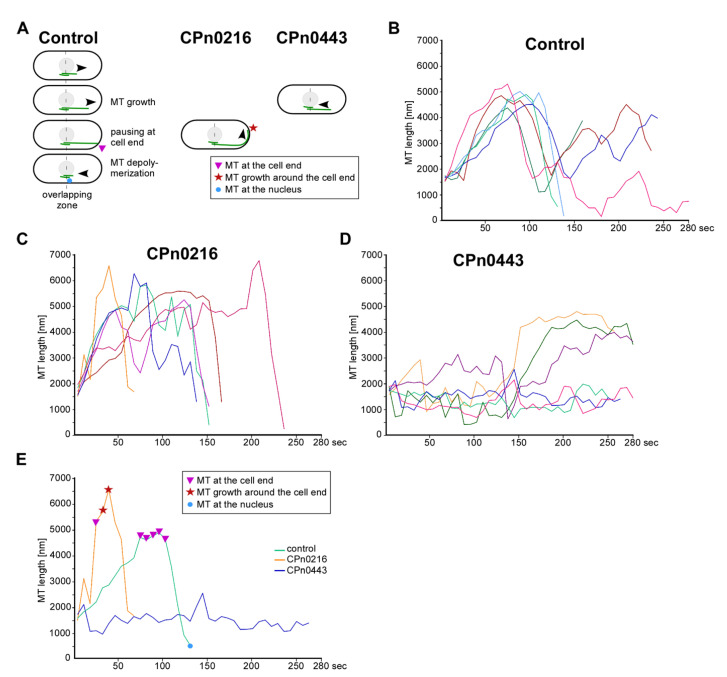
Expression of chlamydial CPn0216 or CPn0443 proteins altered the MT dynamics in yeast. (**A**) Diagrammatic representation of wild-type MT dynamics of an *S. pombe* interphase cell (control cells) and the main differences observed in yeast transformants that expressed CPn0216 or CPn0443. Interphase MTs from control cells polymerized from the vicinity of the nucleus in an oriented manner along the long cell axis to the cell end, then paused (pink triangle) and depolymerized back to the nucleus (blue dot). MTs of CPn0216 expressing cells did not stop polymerization once the cell end was reached but continued to grow around the cell end, resulting in a “curling” phenotype (red star), while the MTs of CPn0443 expressing cells depolymerize before reaching the cell end. (**B**–**D**) Examples of the dynamic behavior of individual interphase MTs from the control, CPn0216- or CPn0443-expressing cells that demonstrated consistently aberrant MT dynamics caused by the expression of a chlamydial protein. Each color represents MT dynamics for individual MTs from cells transformed with the indicated plasmid. (**E**) Representative examples of the dynamic behavior of a single MT from either a control, CPn0216- or CPn0443-expressing cell. The MT dynamics of MTs that were 1.5–2 µm in length (from the middle of the MT overlap zone at the nucleus) were measured via live-cell imaging of GFP-α-tubulin-expressing transformants.

We chose transformants that expressed either CPn0216 or CPn0443 for further analysis, as these cells showed the most aberrant MT cytoskeleton: *cpn0216* expression led to abnormally long MTs that continued to grow once the cell tip was reached, while *cpn0443* expression resulted in aberrantly short MTs (Movies S1–S3). Using live-cell imaging of GFP-α-tubulin transformants, MTs of the control cells polymerized from the vicinity of the nucleus in an oriented manner along the long cell axis to the cell end, followed by pausing and depolymerizing back to the nucleus (diagrammatically shown in Figure 5A and Movie S1). This type of MT dynamic pattern occurred many times during the interphase. Individual dynamics of MT bundles of control transformants are shown in Figure 5B. In contrast, MTs of transformants expressing CPn0216 showed an aberrant and much more diverse phenotype, with the most severe being MT bundles that polymerized faster than in the wild-type and did not pause upon reaching the cell end. Instead, these MT-plus ends continued to grow, resulting in curved MTs before catastrophe (Figure 5C and comparison with the control in Figure 5E; Movie S2). In contrast, CPn0443-affected MTs did not reach the cell tip but showed multiple rounds of polymerization and depolymerization events resulting in aberrantly short MTs (Figure 5D and comparison with the control in Figure 5E; Movie S3).

### 2.6. CPn0443 Associated with MTs in Different Cell Systems

**Figure 6:**
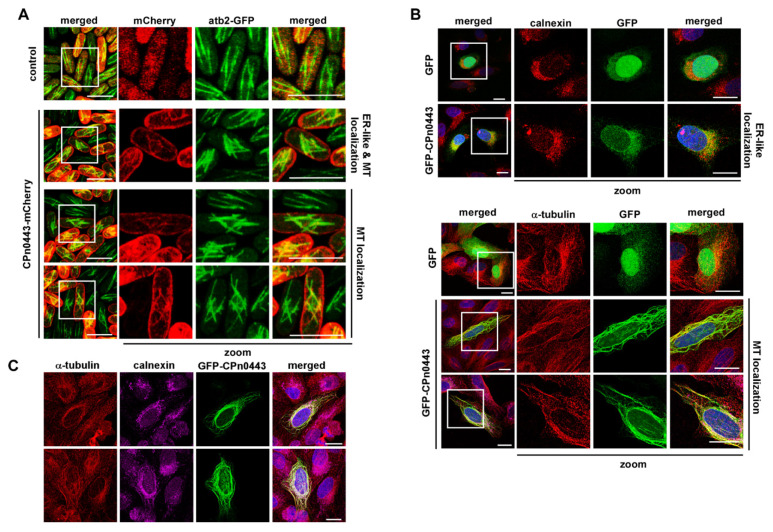
Ectopically expressed CPn0443 localized with the MT cytoskeleton and the ER. (**A**) Photomicrographs of GFP-α-tubulin-expressing (green) *S. pombe* cells (*nmt81::GFP-atb2^+^*). Cells were transformed with plasmids expressing mCherry (control) or CPn0443-mCherry. CPn0443-mCherry (red) co-localized with ER-like structures and partially with MTs (green). White boxes show the areas of enlargement; scale bar: 10 µm. Examples shown are representative cells from 3 independent experiments analyzing 50 cells/experiment. (**B**) Confocal images of fixed human U2OS cells that expressed either *gfp* or *gfp-cpn0443*. *gfp-cpn0443*-expressing cells that showed an ER or MT co-localization. ER was visualized with the anti-calnexin antibody (red), MTs with the anti-α-tubulin antibody (red), and DNA with DAPI (blue). White boxes show enlargement (zoom). The examples shown are representative of two independent experiments that analyzed 50 transfected cells/experiment. (**C**) Fluorescent microscopy images of fixed U2OS cells that expressed *gfp-cpn0433* showing co-localization with altered MTs and an altered ER. MTs were visualized with the anti-α-tubulin antibody (red), ER with the anti-calnexin antibody (magenta) and DNA with DAPI (blue). (**B**,**C)** scale bars: 10 µm.

Next, we determined whether the phenotype observed upon CPn0443 expression was caused by CPn0443 binding to MTs. CPn0443 has a moderate overall protein identity of 35% for *Ctr* CT005/IncV. IncV is located in the inclusion membrane and tethers this to the endoplasmic reticulum (ER) to form membrane contact sites (MCS), which are crucial for nonvesicular trafficking-based interorganelle communication [59]. To determine the subcellular localization of CPn0443, we expressed plasmid-encoded tagged variants in yeast and human cells. Microscopic analysis of CPn0443-mCherry-expressing yeast cells showed aberrantly short interphase MTs, as seen when the untagged CPn0443 was expressed, confirming that the tagged protein was functional (Figure 6A). The predominant localization of this chlamydial protein in *S. pombe* was ER-like, with 68.7% of cells analyzed showing this localization [60] (Figure 6A, second row). As short MTs were present close to the nucleus, we also observed some co-staining with MT structures. A subpopulation of CPn0443-mCherry-expressing yeast cells (31.3% of all analyzed cells) showed a clear MT colocalization (Figure 6A, rows three and four)). We concluded that in *S. pombe*, CPn0443 can associate with a subpopulation of MTs.

Next, we determined the CPn0443 localization in human epithelial U2OS cells. For this analysis, U2OS cells were transfected for 18 h with a plasmid containing *gfp*-tagged *cpn0443*. Interestingly, we found results similar to ours for the fission yeast, namely, that 75% of CPn0443-expressing cells showed co-localization of the chlamydial protein with the ER (visualized via calnexin staining) (Figure 6B, top panels). In the remaining 25% of CPn0443-expressing cells, the chlamydial protein was associated with the MT network (Figure 6B, bottom panels). MTs with CPn0443-GFP association showed different degrees of an aberrant MT cytoskeleton, including thick, cable-like MTs that were probably due to varying CPn0443-GFP expression levels. Surprisingly, 33% of cells with CPn0443-GFP that co-localized with MTs also showed calnexin co-localization with MT (Figure 6C). In such cases, MTs were cable-like, which was reminiscent of the overexpression of known microtubule regulators [61,62,63].

### 2.7. CPn0443 Had Microtubule-Binding Activity

**Figure 7:**
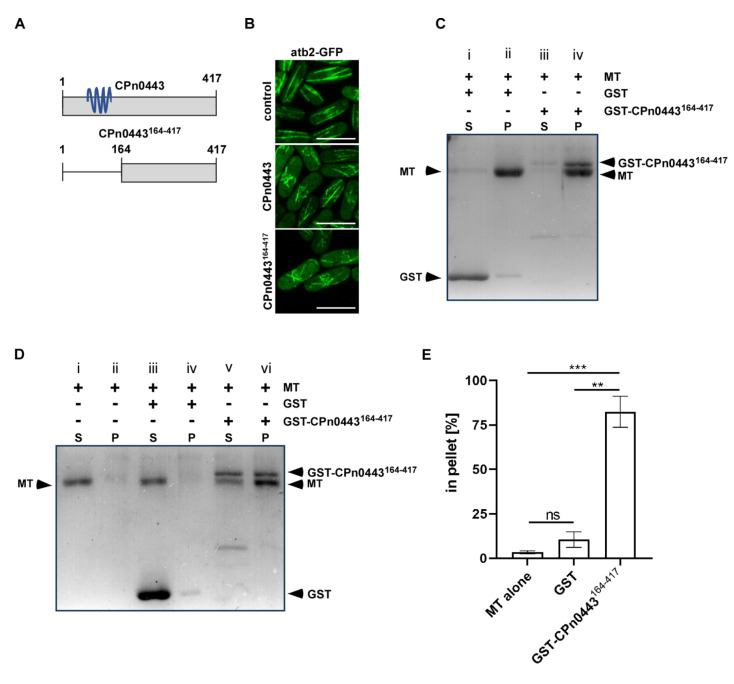
CPn0443 bound MTs. (**A**) Schematic illustration of the CPn0443 full-length protein and CPn0443^164-417^ variant with deletion of the N-terminus. Transmembrane domains are shown in blue. (**B**) Photomicrographs of GFP-α-tubulin-expressing (green) *S. pombe* cells (*nmt81::GFP-atb2^+^*) expressing either CPn0443 or the variant CPn0443^164-417^. Expressions of both CPn0043 and CPn0443^164-417^ gave rise to abnormally short MTs compared with the control cells. (**C**) A representative MT co-sedimentation assay showing a direct interaction between GST-CPn0443^164-417^ and MTs. Bacterially expressed recombinant GST or GST-CPn0443^164-417^ and porcine-derived Taxol-stabilized MTs were incubated at RT followed via high-speed centrifugation (100,000× *g*). Supernatant (S) and pellet (P) fractions were separated via a 15% SDS-PAGE followed by Coomassie staining. Matching control samples without MTs are found in Supplementary Figure S4B. (**D**) SDS gel analysis of the formation of higher-order MT structures was performed in the presence of GST or GST-CPn0443^164-417^ and Taxol-stabilized MTs. After low-speed centrifugation (4000× *g*), equivalent amounts of pellet and supernatant were processed as in (**C**). (**E**) Quantification of the MTs found in pellet fractions shown in (**D**); ns, not significant; *p* < 0.0018 (**), *p* < 0.0008 (***). Results represent typical examples from three independent experiments. Error bars denote ±SEM. Two-tailed unpaired Student’s *t*-test was used to determine statistical significance.

We then explored the possibility that the observed MT defects in CPn0443-expressing cells were a result of direct MT binding by this chlamydial effector protein. Considering that the expression of full-length CPn0443 or CPn0443^164-417^ resulted in comparable microtubule defects in *S. pombe* (Figure 7A,B), the MT regulatory domain was likely to be present in the smaller CPn0443^164-417^ variant. As we were unable to purify the full-length GST-tagged protein, we purified GST-CPn0443^164-417^ and performed MT co-sedimentation assays with Taxol-stabilized MTs [64]. After incubation at RT, MTs and purified proteins were ultra-centrifugated at 100,000× *g* under conditions that are sufficient for the sedimentation of large proteinaceous structures, such as MTs. Under these conditions, the vast majority of a GST control protein remained in the soluble fraction (Figure 7C-i) and only a small percentage co-sedimented with MTs in the pellet fraction (Figure 7C-ii). However, GST-CPn0443^164-417^ was almost entirely found in the pellet fraction (Figure 7C-iv) together with MTs, demonstrating that GST-CPn0443^164-417^ binds MTs directly (Figure 7C, Figure S4B).

We next investigated whether GST-CPn0443^164-417^ was able to modify or aggregate MTs. We took advantage of differential centrifugation and its ability to separate regular MT cylinders from larger macromolecular structures produced via MT bundling. Following a 4000× *g* centrifugation, MTs were found in the soluble fraction when incubated with a buffer (Figure 7D-i) or a GST control (Figure 7D-iii). However, incubation with GST-CPn0443^164-417^ generated larger MT structures that were predominantly found in the pellet fraction (Figure 7D-vi). Additionally, GST-CPn0443^164-417^ was also able to generate large MT structures that were even pelleted at 100× *g* (Figure S4A), similar to known MT-binding proteins that were proposed to bundle MTs [65].

### 2.8. Ectopically Expressed CPn0443 Attenuated Cpn Infection

**Figure 8:**
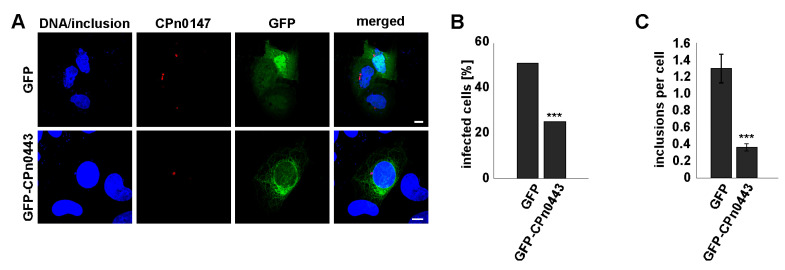
Ectopically expressed CPn0443 resulted in reduced *Cpn* infection. (**A**) Confocal fluorescence images of GFP or GFP-CPn0443-expressing (green) U2OS cells followed by *Cpn* infection (MOI 1) for 30 h. The chlamydial inclusion membrane was visualized with the anti-CPn0147 antibody (red) and DNA/inclusions with DAPI (blue). Scale bars: 5 µm. (**B**,**C**) Quantification of the number of infected cells (**B**) and chlamydial inclusions (**C**) per cell. U2OS cells transfected with GFP or GFP-CPn0443 and infected with EBs were analyzed. Error bars denote ± SEM. Two-tailed unpaired Student’s *t*-test; *p* < 0.001 (***). n = 2; 50 cells per experiment and plasmid.

To determine whether ectopically expressed CPn0443 affected the *C. pneumoniae* infection efficiency, we infected *gfp* (control) or *cpn0443-gfp*-expressing U2OS cells with *Cpn* EBs for 30 h (Figure 8A). We observed a 26% reduction in *Cpn*-infected *cpn0443-gfp*-expressing cells in comparison to the control (Figure 8B). Furthermore, the number of inclusions per cell was significantly decreased compared with the control cells (Figure 7C). This demonstrated that *cpn0443*-expression had a negative impact on *Cpn* infection in mammalian cells.

## 3. Discussion

### 3.1. The MT Cytoskeleton Played a Vital Role in C. pneumoniae Infection

The fission yeast *Schizosaccharomyces pombe* is one of two model yeasts that were instrumental in discovering fundamental eukaryotic processes, such as the cell cycle, and were used as models of major human diseases [66,67,68]. As *S. pombe* has a very distinct interphase MT cytoskeleton, we used this yeast to identify *C. pneumoniae* proteins that manipulate the host cytoskeleton. *C. pneumoniae* is an obligate intracellular human pathogen that is very difficult to manipulate genetically, and thus, the *S. pombe* system has been an excellent tool to identify chlamydial MT modulators. The role of the MT cytoskeleton in bacterial infection processes had not yet been studied systematically, and thus, our analysis uncovered the important role of this cytoskeleton for a bacterial pathogen. Unexpectedly, of the 116 chlamydial proteins tested, which comprised more than 10% of the reduced *C. pneumoniae* genome, we found that 13 massively changed the yeast interphase cytoskeleton. It was possible that the 18 chlamydial proteins found in the yeast screen in category 2 also contributed to the manipulation of the host MT cytoskeleton, but this could not be assayed as the expression in yeast was lethal. Furthermore, although the basic principles between the yeast and the human MT cytoskeleton were conserved, *S. pombe* tubulin does not have all the modifications found in higher eukaryotes. For example, tubulin detyrosination does not appear to exist in *S. pombe*, while *C. trachomatis* stabilizes the MTs surrounding the inclusion via tubulin modifications [25,34,69,70]. This suggests that our screen was highly valuable in identifying MT-modulating proteins, but probably underestimated the number of *Cpn* proteins involved in MT modulation. We concluded that the contribution of the MT cytoskeleton in bacterial pathogenesis was vastly underrated.

### 3.2. The Identified MT-Modulating Proteins Were Expressed at Different Stages of the Chlamydial Developmental Cycle

None of the 13 candidates identified had been analyzed to date, and thus, the mechanisms by which *Cpn* targets aspects of MT biology during infection were unknown. However, transcriptome analysis data exist for all 13 candidates pinpointing to MT manipulation during the entire chlamydial life cycle [48,71,72]. Their expression profiles can be clustered into three classes: “early”, “mid” and “late” (adapted from [48]) (Figure S5). The six early genes, namely, *cpn0284*, *cpn0372*, *cpn0443*, *cpn0565*, *cpn0821* and *cpn1027*, are expressed at high levels already at 6 h post-infection (p.i.), implying that the corresponding proteins are functional at the early stage of infection. Thus, they might be required for establishing the infectious niche, localizing the inclusion in proximity to the nucleus and/or differentiating EBs from RBs. The four mid genes, namely, *cpn0045*, *cpn0186*, *cpn0312* and *cpn0367*, are expressed constitutively between 24 h and 48 h p.i., and thus, these proteins might play a role in RB cell division, immunity evasion or anti-apoptosis. The three late genes, namely *cpn0065*, *cpn0216* and *cpn0365*, have increased expression from 36 h p.i. (Figure S5) and their function could be linked to RB to EB re-differentiation, host cell exit or as EB effector proteins for the new infection.

### 3.3. Eleven of the Identified Proteins Were Inc Proteins

Effector proteins interacting with the host cell MT system need to be associated with the inclusion membrane or secreted into the host cell cytosol. Although our initial selection of chlamydial proteins included several predicted Inc proteins, and thus, was not random, it is noteworthy that 11/13 proteins were putative Inc proteins. To date, eight of these proteins have been analyzed for a functional N-terminal type-3-secretion signal (T3SS) and all of them were able to allow for the secretion of a reporter in the heterologous *Shigella* test system, which would allow for the secretion of these proteins during infection ([39] and references in there). For four of our T3SS-carrying proteins, a subcellular localization during infection was determined: CPn0186 and CPn1027 are associated with the inclusion membrane, while CPn0284 and CPn0357 are bacteria-associated [39,73,74]. It is possible that the two bacteria-associated proteins are secreted at low levels and that the microscopy only detected the bacterial cytosolic protein pool, but not the final subcellular localization.

### 3.4. Inc Proteins and Modulation of the MT Cytoskeleton

How then could the inclusion membrane proteins modulate the host MT cytoskeleton? Analysis of *Ctr* inclusion membrane points to the existence of inclusion membrane microdomains that interact with the MT network/MT-associated proteins [30,31]. Thus, it is possible that the identified Inc proteins interact with different components of the MT cytoskeleton in time and space during the infection cycle. How they crosstalk with the host MT, whether they act in concert and the outcome of the specific MT modulation for infection is at present unclear. However, the MT phenotype observed in yeast cells expressing such proteins might help in defining their function. *cpn0216* belongs to the late expressed genes (Figure S5) and the CPn0216 protein might therefore be required for the early steps in *Cpn* infection, which we showed required an intact MT cytoskeleton (Figure 1). CPn0216-expressing yeast cells showed abnormally long MTs that continued to grow although they have reached the cell end (Figure 5). Thus, the normal MT length control, which is required for numerous cellular functions, was altered by CPn0216. In *S. pombe*, the switch from MT polymerization to depolymerization once MT bundles reach the cell end is accomplished by two spatially distant competitor complexes: the stabilizing Mal3 (EB1 family)-containing complex and the destabilizing Kinesin-8-containing complex [75]. As CPn0216 can also exert its function in yeast cells without the stabilizing complex, we propose that CPn0572 antagonizes an MT destabilizer motor protein to modulate the MT cytoskeleton.

The second protein that we analyzed in more detail, as far as the MT-modulating function is concerned, was CPn0443. CPn0443 is an early expressed protein. This chlamydial protein can bind directly to Taxol-stabilized MTs in vitro and bundle them. In vivo, we found the protein to be associated with the ER and part of the MT cytoskeleton in both yeast and human cells. Interestingly, the shape of the ER, which is MT-associated and contacts numerous organelles, was altered in CPn0443-expressing cells, as demonstrated by calnexin staining (Figure 6C, transfected versus non-transfected cells). The putative *Ctr* homolog of CPn0443, namely, IncV, was shown to link the chlamydial inclusion with the ER [76]. As ER proteins, such as p180, kinectin and CLIMP63, associate with specific MT populations and regulate ER positioning and organelle reallocation [77], we speculate that CPn0443 might mimic such a function.

In summary, our screen in *S. pombe* was the first systematic analysis to evaluate the importance of the host MT cytoskeleton for infection by a bacterial pathogen. We identified 13 uncharacterized *C. pneumoniae* proteins that were expressed at different stages of the chlamydial infection cycle and these all significantly altered the MT cytoskeleton. As this obligate intracellular pathogen has a highly reduced genome, this work uncovered a hitherto vastly underestimated role for this dynamic cytoskeleton in bacterial pathogenesis.

## 4. Material and Methods

### 4.1. Yeast Strains, Media and Growth Conditions

*S. pombe* strains used:

h^−^ his3-D1 ade6-M210 leu1-32 ura4-D18 (wild-type strain); h^+^ kan^R^::nmt81::GFP-atb2^+^ ade6-M210 his3D1 leu1-32 ura4-D18; h^+^ mal3-pk-GFP::ura4^+^ ade6-M216 ura4-D18 leu1-32 his3-D1; h^+^ nda2-KM52 his3-D1 ade6-M21 leu1-32 ura4-D18; h^−^ nda3-KM311 ade6-M210 leu1-32 ura4^−^.

*S. pombe* strains were transformed with control plasmids pJR2-3XU or pJR2-3XL [49] or derivatives with a specific *Cpn* gene cloned behind the *nmt1^+^* promoter. Generation of these plasmids was achieved via homologous recombination in *S. cerevisiae* CEN.PK2 [78]. For patch test assays, *S. pombe* transformants were grown in liquid selective minimal medium in the presence or absence of thiamine for 24 h at 25 °C, diluted, and plated on plasmid-selective minimal medium with/without 5 μg/mL thiamine and with/without 3–9 μg/mL TBZ (Merck KGaA, Darmstadt, Germany) or with/without 0.5–4.5 μg/mL of MBC (Merck KGaA, Darmstadt, Germany). At least two independent transformants per plasmid were assayed.

### 4.2. Generation of the Cpn Expression Library for Screening in S. pombe

A total of 116 *Cpn* genes [48,79] were selected for the *Cpn* expression library in *S. pombe*. Genes (lengths between 291–4614 bp) were amplified via PCR using *Cpn GiD* DNA as a template [80] and primers with 20-nucleotide homology to the respective gene and 40-nucleotide homology to the *S. pombe* expression vector pJR2-3XU. Genes were cloned downstream of the *nmt1^+^* promoter using homologous recombination in *S. cerevisiae* CEN.PK2 and grown under plasmid-selective conditions [78]. Plasmids were isolated from *S. cerevisiae* and amplified in *E. coli* XL_1_-blue. Correct insert integration was verified via DNA sequencing. Genes with sequence deviation (nucleotide exchanges or insertions) were *cpn0045*, *cpn0132*, *cpn0150* and *cpn0132*. Cloning of these genes was repeated but subsequent sequence analysis revealed the existence of the sequence deviation in the *Cpn* GiD strain (Supplementary Table S1A) [81].

### 4.3. Yeast Protein Extracts and Western Blot Analysis

Whole yeast protein extracts were obtained as described [82]. Actin and tubulin were visualized on an 8% SDS-PAGE with the anti-actin antibody (Thermo Scientific, Waltham, MA, USA) or anti-α-tubulin antibody (Santa Cruz Biotechnology, Dallas, TX, USA). The densitometry of the bands was determined using Image Lab 6.1 (BioRad, Hercules, CA, USA) and quantification using GraphPad Prism8 (Graph-Pad Software, Inc., Boston, MA, USA).

### 4.4. Mammalian Cell Lines and Production of Infectious Cpn EBs

HEp-2 (ATCC: CCL-23) and U2OS (ATCC: HTB-96) cells were cultured in Dulbecco’s Modified Eagle Medium (Thermo Scientific, Waltham, MA, USA) supplemented with 10% FCS, MEM vitamins and nonessential amino acids (Thermo Scientific, Waltham, MA, USA). *Cpn* GiD was propagated in HEp-2 cells and EBs were purified in a 30% gastrographin solution (Bayer Vital GmbH, Leverkusen, Germany) and stored at −80 °C in an SPG buffer (220 mM sucrose, 3.8 mM KH_2_PO_4_, 10.8 mM Na_2_HPO_4_, 4.9 mM L-glutamine).

### 4.5. Drug-Induced MT-Depolymerization and Chlamydia Infection

HEp-2 cells were treated with 10 µg/mL nocodazole (Merck KGaA, Darmstadt, Germany) in supplemented DMEM medium for 30 min at 37 °C, followed by nocodazole washout via replacement of the medium with fresh DMEM medium containing purified EBs (MOI 5), followed by centrifugation at 2980 rpm at 37 °C for 20 min. The supernatant medium was then replaced with fresh DMEM medium containing 12 µg/mL cycloheximide. Cells were incubated at 37 °C for 30 h. Samples were fixed at specific time points (0–30 h) with 3% paraformaldehyde in 37 °C warm PBS for 10 min, permeabilized with 2% Saponin in PBS and immunostained for microscopy. MTs were visualized with the anti-α-tubulin antibody (OriGene Technologies, Inc., Rockville, MD, USA), the inclusion membrane with an anti-CPn0147 antibody [83] and DNA with DAPI (Merck KGaA, Darmstadt, Germany).

### 4.6. Transfection and Staining of Mammalian Cells

The transfection of U2OS cells was achieved according to the manufacturer’s instructions (Thermo Scientific, Waltham, MA, USA). Transfected samples were fixed 18 h p.i. with fresh, pre-warmed 3% paraformaldehyde in 37 °C warm PBS for 10 min and processed. MTs were visualized with the anti-α-tubulin antibody (OriGene Technologies, Inc., Rockville, MD, USA), endoplasmatic reticulum (ER) with the anti-calnexin antibody (Santa Cruz Biotechnology) and DNA with DAPI (Merck KGaA, Darmstadt, Germany). For a following *Cpn* infection, transfected cells were treated as described above.

### 4.7. Microscopy and Image Processing

Fixed mammalian cells: images were acquired using an inverse Nikon TiE Live Cell Confocal C2plus with a 100-x TIRF objective and a C2 SH C2 Scanner (Nikon Corporation, Tokyo, Japan). All images are displayed as maximum intensity projections, except when noted. Analysis of images and measurements were generated with Nikon Element software and ImageJ 1.47v (National Institutes of Health, Bethesda MD, USA).

Live-cell imaging of *S. pombe* cells: Yeast cells were grown in plasmid-selective minimal media (MM) with supplements [84]. Prior to microscopy, cells were grown at 25 °C for 24 h in liquid MM without thiamine to the early/mid-log phase. Microscopy slides were prepared by mounting cells on agarose pads [85]. Live-cell images were acquired at RT using a Zeiss LSM 880 inverted confocal microscope (Carl Zeiss Microscopy GmbH, Oberkochen, Germany) equipped with an Airyscan detector and a Plan-Apochromat 63x/1.4 oil immersion objective lens. Raw Airyscan images were processed using the ZEN Black software’s Airyscan Processing function. For the observation of MT dynamics, Z-stacks were collected at 7 s intervals and maximum intensity projections were used for analysis using ZEN 3.0 blue edition (Carl Zeiss Microscopy GmbH, Oberkochen, Germany).

For the quantification of MT dynamics, *nmt81::GFP-atb2*^+^ cells of 9 to 12 µm in length were assayed. The antiparallel MT overlapping zones at the nucleus were chosen as the starting point for the measurement and MTs with a length of 1.5–2 µm were chosen for MT dynamic measurements.

### 4.8. MT Binding and Bundling Assays

Recombinant GST and GST-CPn0443^164-417^ were expressed in *E. coli* Rosetta (DE3) cells (Merck KGaA, Darmstadt, Germany) and purified using glutathione agarose beads (Merck KGaA, Darmstadt, Germany) according to manufacturer’s instructions and dialyzed o/n at 4 °C in buffer A1 (50 mM Hepes-HCl pH 7.4, 150 mM NaCl, 20% glycerol *w*/*v*). The purity of GST-CPn0443^164-417^ was analyzed using SDS-PAGE and Coomassie blue staining. Taxol-stabilized MTs were generated in vitro according to the manufacturer’s instructions (Cytoskeleton Inc., Denver, CO, USA). MT binding assays were carried out as follows: 25 µL of Taxol-stabilized MTs (equivalent to 4 µM tubulin dimer) in 80 mM PIPES pH 7.0, 2 mM MgCl_2_, 0.5 mM EGTA, 72 µM GTP, 0.4% glycerol (*w*/*v*) and 18 µM Taxol were incubated with 25 µL of 4.6 µM GST-CPn0443^164-417^ or a GST control in buffer A1 for 30 min at RT. Mixtures were then layered on top of a 100 µL glycerol cushion (80 mM PIPES pH 7.0, 1 mM MgCl_2_, 1 mM EGTA, 60% glycerol, 20 µM Taxol) and centrifuged at 100,000× *g* at RT for 45 min. The supernatant was carefully collected before the removal of the glycerol cushion. Pellets were resuspended and loaded next to their corresponding supernatant fraction on a 15% SDS gel, followed by Coomassie staining. Identification of the formation of larger MT assemblies was achieved following the same procedure, except that the protein mixture was centrifuged at 4000× *g* at RT for 45 min. The densitometry of the bands in soluble and pellet fractions was carried out using Image Lab (BioRad, Hercules, CA, USA), and the values of MTs in pellet fractions were graphed as percentages of total MTs found in soluble and pellet fractions together.

## Data Availability

Data are contained within the article or Supplementary Material.

## Supplementary Materials

The following supporting information can be downloaded at: https://www.mdpi.com/article/10.3390/ijms24087618/s1. References [86,87,88,89] are cited in the Supplementary Materials.
