# Supplementary material for: A Functional Yeast-Based Screen Identifies the Host Microtubule Cytoskeleton as a Target of Numerous Chlamydia pneumoniae Proteins
Source: Int J Mol Sci. 2023 Apr 20;24(8):7618. doi: 10.3390/ijms24087618 (PMC10142024; doi:10.3390/ijms24087618)
Supplement: Supplementary file 1 [file ijms-24-07618-s001.zip › 3 - Supp Figures and movies/Supp Figures Supp FINAL.pdf]

A functional yeast-based screen identifies the host microtubule cytoskeleton as a target of numerous *Chlamydia pneumoniae* proteins

Carolin Wevers [1*], Mona Höhler [1*], Abel R. Alcázar-Román [1], Johannes H. Hegemann [2 #], Ursula Fleig [1 # $]

[1]Eukaryotic Microbiology, Institute of Functional Microbial Genomics, Heinrich-Heine-University, Düsseldorf, Germany.

[2]Institute of Functional Microbial Genomics, Heinrich-Heine-University, Düsseldorf, Germany.

* joint first authors

[#] joint senior authors

[$] Correspondence may be addressed to:
Ursula Fleig; Email: fleigu@hhu.de

Supplementary Tables, Figures and Legends

Table S1. Alterations in DNA sequence of some *Cpn* genes and summary of growth phenotypes of *S. pombe* transformants expressing one of the 116 *Cpn* proteins.

A Genes with sequence deviation leading to altered amino acids. Green: wildtype *Cpn* GiD amino acid sequence; red: aa sequence deviation verified by sequencing. **B** The 116 *Cpn* proteins chosen for analysis were selected as indicated: Inc proteins (i, [1]; ii, [2]), proteins affecting *S. cerevisiae* yeast growth (iii, [3]; iv, [4]) or being highly expressed (v, [5]). Growth phenotypes of transformants expressing one of the 116 *Cpn* genes were grouped into one of the 4 categories defined in Figure. 2C.

A

Cpn gene	Difference to *Cpn* GiD genome	Position in DNA sequence (nt)	*Cpn* GiD aa sequence	Sequenced plasmid DNA sequence (aa)
cpn0045	Insert of 10 nt leads to changed aa sequence	1708	Tyr-Lys-Ser-Gly-Phe-Arg-Val-Stop (TGA)	Tyr-Arg-Arg-Asn-Lys-GluArg-Phe-Gln-Ser-Leu
cpn0132	nt exchange leads to aa exchange	748	Leu-Ser-Glu	Leu-Ala-Glu
cpn0150	nt exchange leads to aa exchange	3973	Asp-Cys-Asp	Asp-Ser-Asp
cpn0912	Insert of 27 nt	133	Ala-Glu	Ala-Asp-Glu-Ile-Gln-Leu-Pro-Ser-Pro-Glu

B

Cpn protein	Category	Ref.	*Cpn* protein	Category	Ref.
CPn0007	1a	i, ii	CPn0474	2	i, ii
CPn0026	1b	i, ii	CPn0480	2	i, ii
CPn0041	1b	i, ii	CPn0481	2	i
CPn0045*	3a	i, ii	CPn0488	1a	iii
CPn0061	1a	v	CPn0491	1a	iii
CPn0065	3a	i, ii	CPn0512	1a	v
CPn0066	2	i	CPn0517	1a	i, ii
CPn0067	1b	i, ii	CPn0518	1a	v
CPn0107	1b	v	CPn0523	1a	i, ii
CPn0124	1a	i, ii	CPn0524	1a	i, ii
CPn0126	1a	i, ii	CPn0525	1a	iii
CPn0129	2	ii	CPn0537	1a	i
CPn0131	1a	i, ii	CPn0544	1a	iii
CPn0132*	1a	i, ii	CPn0554	1a	i, ii
CPn0147	1a	i, ii	CPn0556	1a	i, ii
CPn0150*	1a	i, ii	CPn0565	3a	i, ii
CPn0157	1a	i	CPn0585	1a	i, ii
CPn0164	1a	i, ii	CPn0592	1a	iii
CPn0166	1a	i, ii	CPn0601	1a	i, ii
CPn0169	1a	i, ii	CPn0602	1a	i, ii
CPn0173	1b	i, ii	CPn0648	2	iii
CPn0186	4	i, ii	CPn0658	1a	v
CPn0203	1b	i	CPn0671	1b	v
CPn0212	1b	i, ii	CPn0729	1a	iii
CPn0214	1a	i, ii	CPn0746	1a	v
CPn0215	1a	i, ii	CPn0753	2	i, ii
CPn0216	4	i, ii	CPn0755	1b	i, ii
CPn0218	1a	ii	CPn0770	2	i, ii
CPn0241	2	ii	CPn0813	1a	iv
CPn0242	1a	i, ii	CPn0815	1a	iv
CPn0255	1a	iii	CPn0821	4	iii
CPn0262	1a	v	CPn0829	1b	i
CPn0284	3b	i, ii	CPn0830	2	i
CPn0285	2	i, ii	CPn0834	2	i
CPn0288	1a	i, ii	CPn0844	1a	v
CPn0291	1b	i, ii	CPn0850	1b	iv
CPn0292	1a	i, ii	CPn0852	1a	v
CPn0308	2	i, ii	CPn0869	1a	ii
CPn0312	3b	i, ii	CPn0879	1a	v
CPn0350	2	iii	CPn0906	1a	v
CPn0357	3b	i, ii	CPn0909	1a	v
CPn0365	4	i, ii	CPn0912*	1a	v
CPn0366	1a	i, ii	CPn0930	1a	i, ii
CPn0370	2	i, ii	CPn0938	2	i, ii
CPn0371	1b	i, ii	CPn0966	1a	iv
CPn0372	3a	i, ii	CPn0994	1a	i
CPn0381	1a	i	CPn0998	2	v
CPn0404	1a	i	CPn1003	1a	ii
CPn0409	1a	v	CPn1008	1a	i, ii
CPn0431	1a	i, ii	CPn1027	4	i
CPn0432	1b	i, ii	CPn1029	1b	i, ii
CPn0440	1a	i, ii	CPn1046	1a	iv
CPn0441	1b	iii	CPn1051	1a	i, ii
CPn0442	2	i, ii	CPn1054	1a	i, ii
CPn0443	4	i, ii	CPn1055	1a	i, ii
CPn0456	1a	iv	CPn1060	1a	iv
CPn0465	1b	v	CPn1062	1a	iv
CPn0467	1b	iv	CPn1070	1a	iv

Table S2. Overview of the 13 *Cpn* proteins identified to modulate MTs.

Characteristics of the 13 *Cpn* proteins identified in the screen include: aa length, localization of transmembrane domains (TM), predicted function and validated localization. Putative *Ctr* homologs are shown as are homologs from other chlamydia species. Inc: inclusion membrane protein; X: no homologue identified. i, [1]; ii, [6]; iii, [7]; iv, [8]; v, [9]; vi, [10]. For transmembrane domain prediction "Polyphobius prediction" was used and for protein identities PRALINE sequence alignment was used.

Chlamydia pneumoniae						*Chlamydia trachomatis*		Identity [%] between *Cpn* and *Ctr* proteins			
Protein name	length [aa]	TMs[1] (aa)	Localization		Literature	Predicted Homologue	length [aa]	Whole protein[2]	C-Term behind TMs[2]	Protein without TMs[2]	Conserved in other Chlamydia
			Predicted	Validated							
CPn0045	574	(29-47)	Inc	X	i	X	X	X	X	X	X
CPn0065	576	(32-55); (61-86); (243-263); (269-291)	Inc	X	i	CT288 [iv]	563	24	24	26	C. muridarum
CPn0186	390	(38-62); (68-88)	Inc	inclusion	i, ii	CT119 / IncA	273	28	28	29	X
CPn0216	145	(25-51); (57-81)	Inc	X	i	X	X	X	X	X	X
CPn0284	165	(33-56); (58-82)	Inc	bacteria	i, vi	X	X	X	X	X	X
CPn0312	151	(7-26); (38-56); (95-112); (118-137)	Inc	X	i	CT101 [iv]	153	33	X	9	C. abortus (CAB456)
CPn0357	283	(27-52); (57-76)	Inc	bacteria	i	X	X	X	X	X	DUF1389 in C. pecorum, C. psittaci, C. abortus
CPn0365	339	(33-54); (60-79)	Inc	X	i	X	X	X	X	X	C. pecorum
CPn0372	105	(36-62); (68-90)	Inc	X	i	X	X	X	X	X	X
CPn0443	417	(53-74); (80-102); (114-138); (144-165)	Inc	X	i	CT005 / IncV [iv]	363	35	25	29	C. muridarum, C. Caviae, C. psittaci, C. abortus, C. pecorum
CPn0565	366	(39-58); (64-85)	Inc	X	i	CT449 [iv]	110	34*	X	X	C. abortus, C. pecorum, C. psittaci, C. caviae
CPn0821	334	X	unknown	X		CT566 [v]	330	52	X	X	C. caviae, C. psittaci, C. abortus
CPn1027	527	(34-58); (64-89)	Inc	inclusion	i, iii	X	X	X	X	X	X

X = no data
[1] Polyphobius Prediction
[2] PRALINE Sequence alignment

* Alignemnt with the first 110 aa
i Dehoux et al. 2011 doi: 10.1186/1471-2164-12-109
ii Subtil et al. 2001 https://doi.org/10.1046/j.1365-2958.2001.02272.x
iii Flores et al. 2007 https://doi.org/10.1099/mic.0.2006/002956-0
iv Bannantine et al. 2000 DOI: 10.1046/j.1462-5822.2000.00029.x
v Subtil et al 2005 doi:10.1111/j.1365-2958.2005.04647.x
vi Luo et al. 2007 doi: 10.1186/1471-2180-7-38

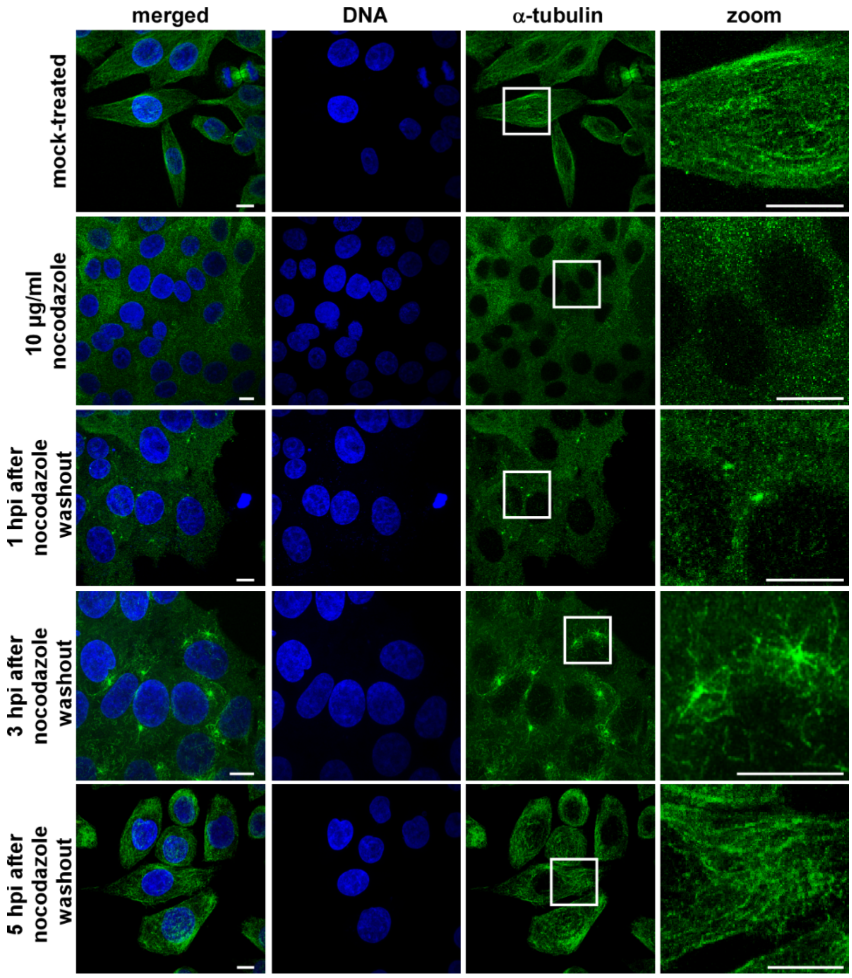

Figure S1. MT de-polymerization followed by re-polymerization after nocodazole removal.

Confocal images of mock-treated (upper panels) or nocodazole-treated HEp-2 cells. Cells were incubated with 10 µg/ml nocodazole for 30 min at 37°C leading to a disassembled MT cytoskeleton (2nd lane from top). Nocodazole washout was performed via media exchange. Re-formation of the MT cytoskeleton was monitored after nocodazole washout and was complete after 5 hrs. MTs were visualized with anti-α-tubulin antibody (green) in fixed cells and DNA with DAPI (blue). White boxes show enlargements. Scale bars, 10 µm.

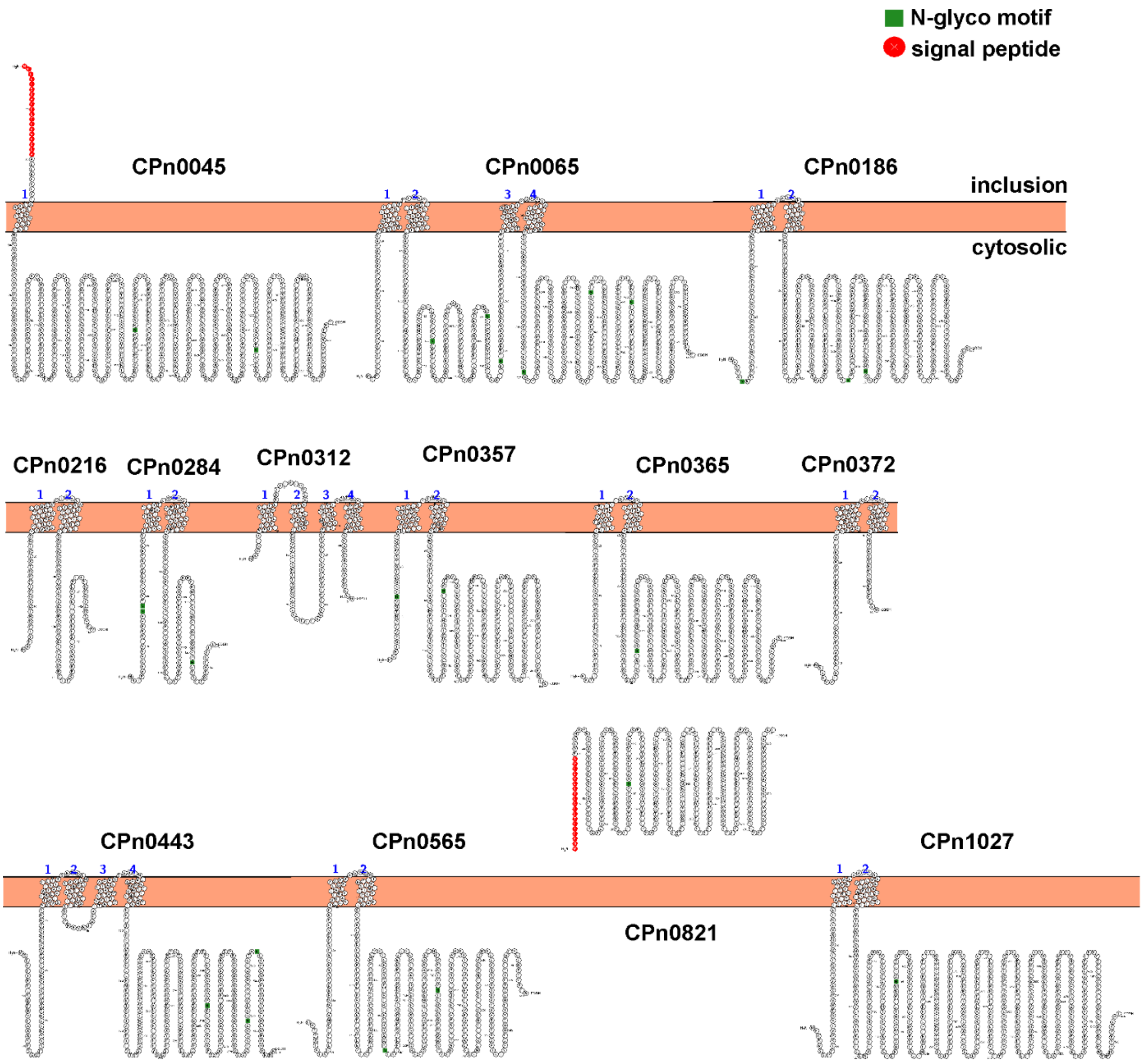

Figure S2. Schematic illustration of the 13 CPn proteins and the localization of the transmembrane domains. Single illustrations were created using Protter. Signal sequences are in red; N-glyco motifs in green.

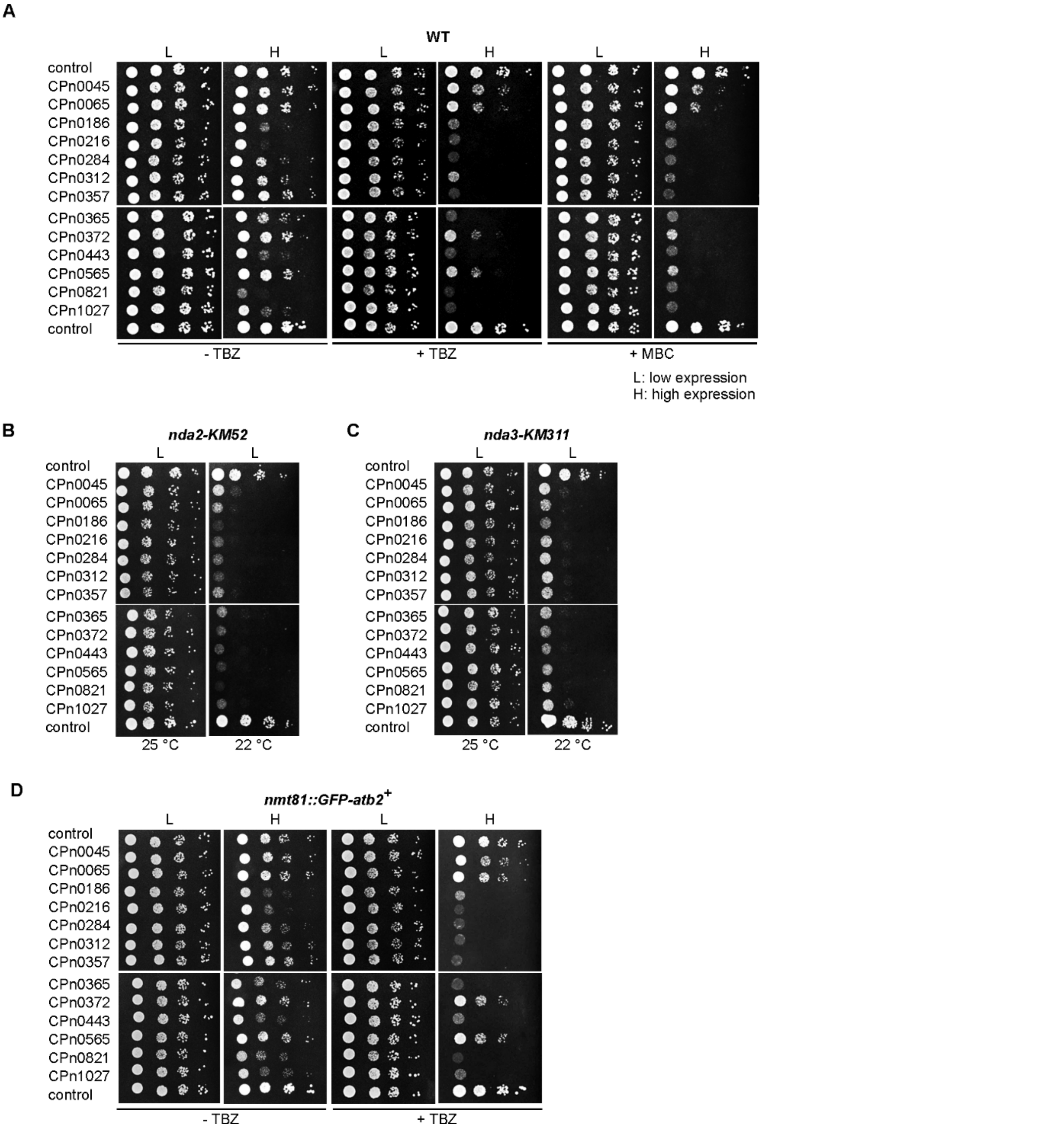

Figure S3. Yeast growth phenotypes caused by expression of *Cpn* proteins.

A Serial dilution patch tests (10^4-10^1 cells) of a wildtype (WT) yeast strain expressing either a control plasmid or one of 13 plasmids with the indicated *Cpn* gene (systematic names on the left). *S. pombe* transformants were grown for 5 days at 25°C on plasmid-selective media without (-TBZ and -MBC) or with (+TBZ and +MBC). Concentrations used: 7 µg/ml TBZ or 2.5 µg/ml MBC; L and H: low or high expression of relevant *Cpn* gene. **B and C** Serial dilution patch tests of cold-sensitive *nda2-KM52* and *nda3-KM311* strains transformed with a control plasmid or expressing the indicated *Cpn* genes. Cells were incubated at 25 °C or 22 °C for 6 days. The non-permissive temperature for the tubulin mutant strains is 20 °C. L; low expression. **D** Serial dilution patch tests of *nmt81::GFP-atb2*$^+$ *(GFP-α-tubulin)* transformants. Cells were incubated at 25 °C for 6 days without or with 6 µg/ml TBZ, L; low expression, H; high expression.

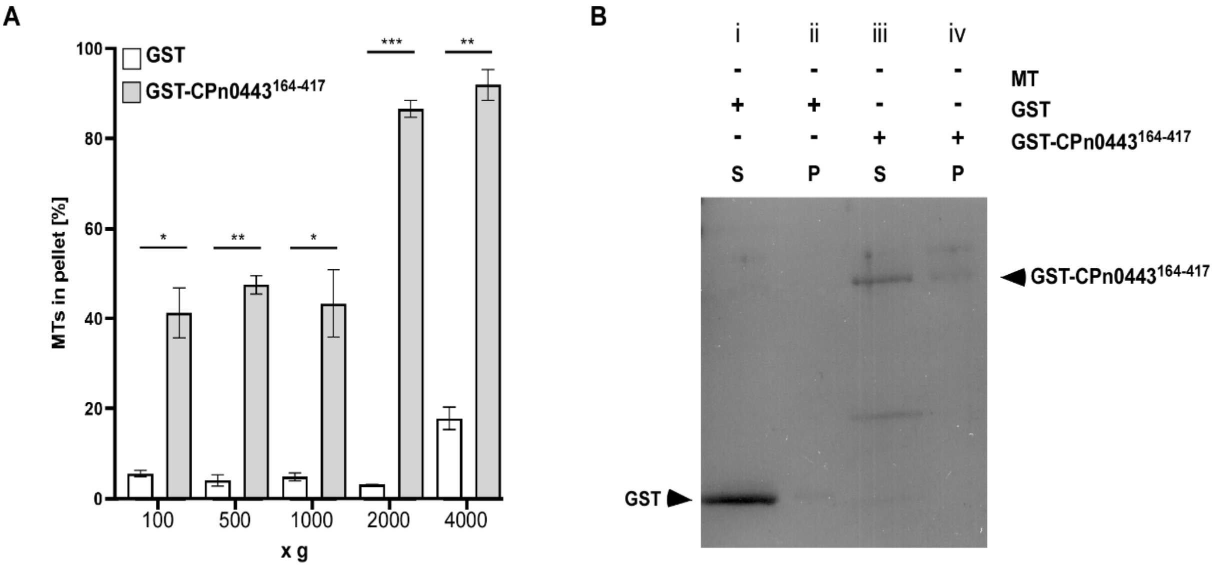

Figure S4. GST-CPn0443$^{164-417}$ forms macromolecular MT structures

A SDS gel analysis of GST-CPn0443$^{164-417}$-derived higher-order MT structures was performed as in Figure 7C at different centrifugal speeds. Quantification of the pellet fractions are shown. Error bars denote ±SEM, n=2 experiments. Two-tailed student's t-test was used to determine statistical significance: 100 xg: $p<0.0239$ (*), 500 xg: $p<0.0021$ (**), 1000 xg: $p<0.0345$ (*), 2000 xg: $p<0.005$ (***), 4000 xg: $p<0.0019$ (**). **B** Representative co-sedimentation assay of GST and GST-CPn0443$^{164-417}$ showing that both proteins predominantly stay in the supernatant fraction in the absence of MTs, while in the presence of MTs (Figure 7C) they are found in the pellet fraction.

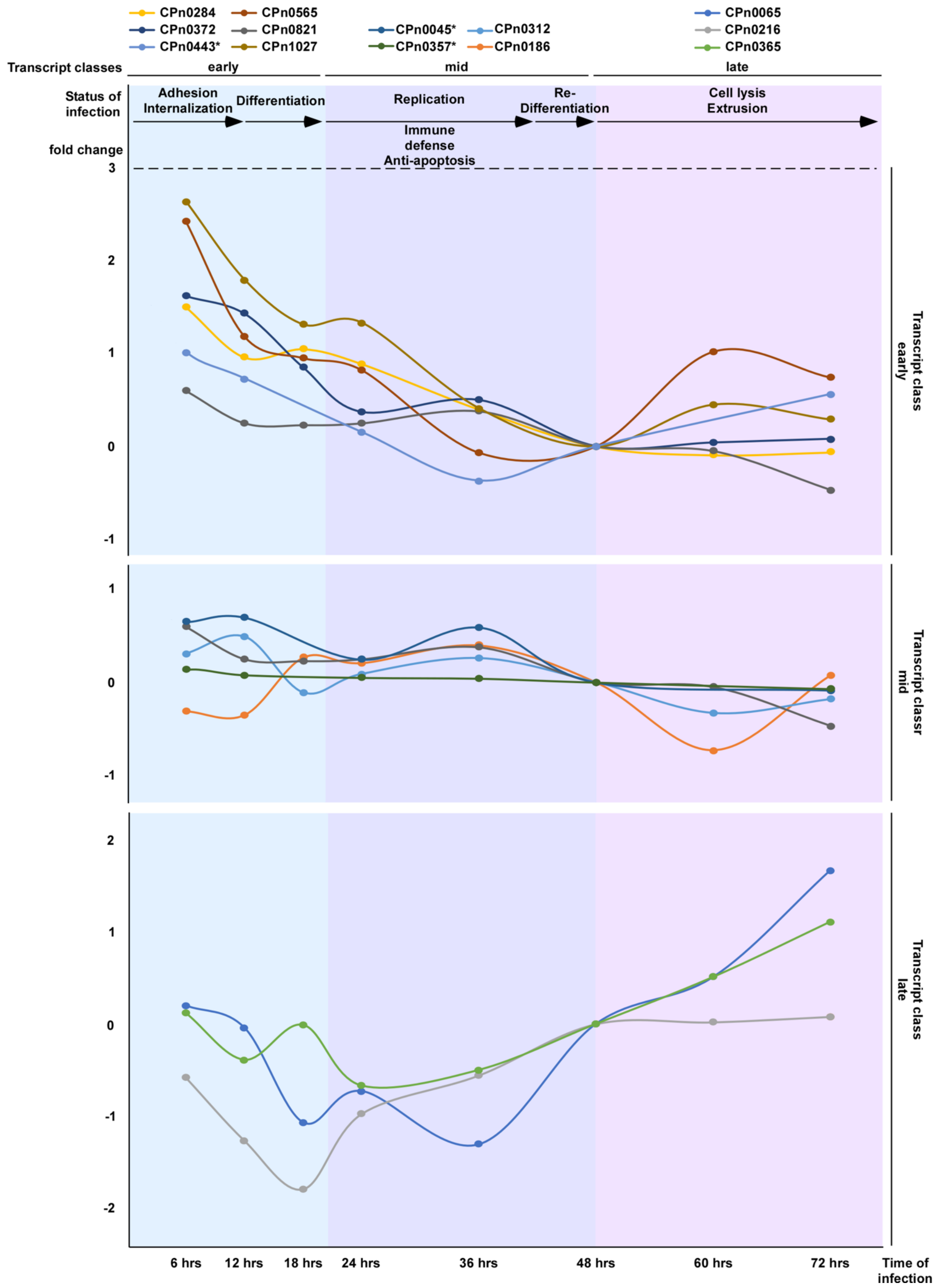

Figure S5. Graphical visualization of the transcriptomes of the 13 Cpn proteins.

The transcriptome data were taken from [5] except for the proteins marked with *. These come from an unpublished transcriptome analysis from the Hegemann lab [11]. Transcripts were categorized as early, mid or late .

Supplementary Movie Legends

Movie 1 Live cell images of *nmt81::GFP-atb2*$^{+}$ cells transformed with a control plasmid. 7 seconds intervals, total 280 seconds. Scale bar, 5 μm.

Movie 2 Live cell images of *cpn0216*-expressing *nmt81::GFP-atb2*$^{+}$ cells 7 seconds intervals, total 280 seconds.

Movie 3 Live cell images of *cpn0443*-expressing *nmt81::GFP-atb2*$^{+}$ cells 7 seconds intervals, total 280 seconds.

1. Dehoux, P.; Flores, R.; Dauga, C.; Zhong, G.; Subtil, A. Multi-genome identification and characterization of chlamydiae-specific type III secretion substrates: the Inc proteins. *BMC Genomics* **2011**, *12*, 109, doi:10.1186/1471-2164-12-109.
2. Lutter, E.I.; Martens, C.; Hackstadt, T. Evolution and conservation of predicted inclusion membrane proteins in chlamydiae. *Comp Funct Genomics* **2012**, *2012*, 362104, doi:10.1155/2012/362104.
3. Sisko, J.L.; Spaeth, K.; Kumar, Y.; Valdivia, R.H. Multifunctional analysis of Chlamydia-specific genes in a yeast expression system. *Mol Microbiol* **2006**, *60*, 51-66.
4. Herbst, F. Identifizierung und Charakterisierung potentieller neuer Effektorproteine aus Chlamydia pneumoniae. Heinreich-Heine-Universität, Düsseldorf, 2011.
5. Maurer, A.P.; Mehlitz, A.; Mollenkopf, H.J.; Meyer, T.F. Gene expression profiles of Chlamydophila pneumoniae during the developmental cycle and iron depletion-mediated persistence. *PLoS Pathog* **2007**, *3*, e83.
6. Subtil, A.; Parsot, C.; Dautry-Varsat, A. Secretion of predicted Inc proteins of Chlamydia pneumoniae by a heterologous type III machinery. *Mol Microbiol* **2001**, *39*, 792-800.
7. Flores, R.; Luo, J.; Chen, D.; Sturgeon, G.; Shivshankar, P.; Zhong, Y.; Zhong, G. Characterization of the hypothetical protein Cpn1027, a newly identified inclusion membrane protein unique to Chlamydia pneumoniae. *Microbiology (Reading)* **2007**, *153*, 777-786, doi:10.1099/mic.0.2006/002956-0.
8. Bannantine, J.P.; Griffiths, R.S.; Viratyosin, W.; Brown, W.J.; Rockey, D.D. A secondary structure motif predictive of protein localization to the chlamydial inclusion membrane. *Cell Microbiol* **2000**, *2*, 35-47, doi:10.1046/j.1462-5822.2000.00029.x.
9. Subtil, A.; Delevoye, C.; Balana, M.E.; Tastevin, L.; Perrinet, S.; Dautry-Varsat, A. A directed screen for chlamydial proteins secreted by a type III mechanism identifies a translocated protein and numerous other new candidates. *Mol Microbiol* **2005**, *56*, 1636-1647.
10. Luo, J.; Jia, T.; Flores, R.; Chen, D.; Zhong, G. Hypothetical protein Cpn0308 is localized in the Chlamydia pneumoniae inclusion membrane. *Infect Immun* **2007**, *75*, 497-503, doi:IAI.00935-06 [pii]

10.1128/IAI.00935-06.

11. Murra, G. Identifizierung und Charakterisierung von Adhäsionsproteinen des humanpathogenen Erregers Chlamydia pneumoniae

. Heinrich-Heine-Universität Düsseldorf, Düsseldorf, Germany, 2009.
